# Improving adsorption performance of l-ascorbic acid from aqueous solution using magnetic rice husk as an adsorbent: experimental and RSM modeling

**DOI:** 10.1038/s41598-023-38093-x

**Published:** 2023-07-05

**Authors:** Azam Aslani, Hadiseh Masoumi, Hossein Ghanadzadeh Gilani, Ahad Ghaemi

**Affiliations:** 1 Department of Chemical Engineering, University of Guilan, Rasht, 4199613776 Iran; 2 School of Chemical, Petroleum and Gas Engineering, Iran University of Science and Technology, Tehran, 13114-16846 Iran

**Keywords:** Environmental sciences, Chemical engineering

## Abstract

In this research, rice husk (RH) was utilized to prepare a magnetic adsorbent for adsorption of ascorbic acid (AA). The magnetic agent is iron(III) chloride (FeCl_3_). The impact of acid concentration in the range of 400–800 ppm, adsorbent dosage in the range of 0.5–1 g, and contact time in the range of 10–130 min were studied. The Langmuir model had the highest R^2^ of 0.9982, 0.9996, and 0.9985 at the temperature of 15, 25, and 35 °C, respectively, and the q_max_ values in these temperatures have been calculated at 19.157, 31.34, and 38.75 mg/g, respectively. The pseudo-second-order kinetic model had the best agreement with the experimental results. In this kinetic model, the values of q have been measured at 36.496, 45.248, and 49.019 mg/g at the acid concentration of 418, 600, and 718 ppm, respectively. The values of ΔH^o^ and ΔS^o^ were measured 31.972 kJ/mol and 120.253 kJ/mol K, respectively, which proves the endothermic and irregularity nature of the adsorption of AA. Besides, the optimum conditions of the design-expert software have been obtained 486.929 ppm of acid concentration, 0.875 g of the adsorbent dosage, and 105.397 min of the contact time, and the adsorption efficiency in these conditions was determined at 92.94%. The surface area of the RH and modified RH was determined of 98.17 and 120.23 m^2^/g, respectively, which confirms the high surface area of these two adsorbents.

## Introduction

Vitamins have been broadly employed in the pharmaceutical, cosmetic, and food industries^1^. Types of vitamins are divided into two classes in view of their solubility in fat and water. The soluble vitamins in the water are thiamine (B1), riboflavin (B2), niacin (B3), pyridoxine (B6), pantothenic acid (B5), biotin (B7), folic acid (B9), cyanocobalamin (B12) and vitamin C. Vitamin C or l-Ascorbic acid (AA) is one of the imperative vitamins that posse remarkable antioxidant properties which can prevent radical reactions in the body whose damage cells and tissues. Thus, this vitamin can boost the immunity system^2^. Its deficiency can cause a risk of incurable illnesses such as cancer, heart disease, and cataracts^3–5^. Vitamin C is able to prevent microbial activity in food because of having a low-value pH^6^. Nevertheless, the body mechanism of people cannot able to produce vitamin C owing to the absence of l-gluconolactone enzyme and cannot retain this vital agent in the organism. Therefore, adequate content of this vitamin must enter the body through eating some food such as citrus fruits, berries, potatoes, tomatoes, peppers, broccoli, and spinach^7^. Bioactive compounds such as biomolecules used in various industries are often synthetic and are produced during diverse stages of the biotechnology and chemical process. Therefore, separation and purification of this vitamin from an aqueous solution is inevitable.

Diverse separation procedures exist. Each of these procedures has a number of drawbacks and can exert issues during the separation process. For instance, the precipitation procedure cannot able to treat the low concentration of metallic ions, and this method also can widely produce useless materials; microbial electrochemical technology (MET) has a remarkable elimination yield, nevertheless, this method requires a long period of time for eliminating the metallic ions; moreover, the price of the resins in the ion-exchange procedure is exorbitant^8^. Among the separation techniques, adsorption is one of the well-known methods due to the simplicity of performance, tremendous yield, easy recovery, and suitable price. Hence, the batch adsorption method was chosen for this study. Several types of reagents have been exploited as the adsorbent for the removal of metallic ions from contaminated water such as activated carbon^9^, fruit wastes^10^, mineral substances^11–14^, microbes^15^, waste materials^16^, and polymers^17^. In this study, a kind of agricultural waste was used as a natural adsorbent. The annual production of food and crop waste is increasing extensively, thus, it is crucial to manage food waste^18^. For avoiding this challenge, food waste can be converted into beneficial materials. The reuse of agricultural waste is proposed as a convenient and economical approach. Due to the desired performances and the low price of agricultural waste such as banana peel, orange peel, rice husk (RH), tea pulp, walnut shell^19^, Montmorillonite clay^20^, chicken beak^21^, zeolite^22^, and etc., these materials have received much attention. Also, various reagents can be grafted to the structure of the adsorbents for improving their performance such as polymers, metal hydroxide, acids, iron, and other chemical materials like xanthate^23,24^. Some valuable works that demonstrate the superiority of the modified adsorbent over the raw ones are indicated as follows. Foroutan et al. used walnut shell (WSA) and WSA/Starch/Fe_3_O_4_ for the removal of copper ions from the water. The uptake capacity of the copper ions was attained at 29 and 45.4 mg/g for WSA and WSA/Starch/Fe_3_O_4_, respectively^19^. Ahmadi et al. used Montmorillonite clay (MC) and MC/starch/CoFe_2_O_4_ for the removal of methylene blue (MB) and methyl violet (MV) from the wastewater. The uptake capacity of MV using MC and MC/starch/CoFe_2_O_4_ nanocomposite were 29.76 and 43.95 mg/g, respectively, and the uptake sorption capacity of MB using these sorbents were 31.96 and 47.51 mg/g, respectively^20^. Foroutan et al. studied the performance of a chicken beak containing hydroxyapatite (HApB) and modified one with the zeolitic imidazolate framework-8 (ZIF-8) for the removal of nickel ions from the water. The uptake capacity of nickel ions has been obtained at 24.27 and 63.49 mg/g using HApB and HApB/ ZIF-8, respectively^21^. Savari et al. investigated the removal of fluoride from the water using zeolite-zirconium under pulsed and continuous sonication. The uptake capacity of the fluoride has been calculated at 32.98 and 31.73 mg/g at the pulsed and continuous situations, respectively^22^. It was concluded that the modification with the magnetic particles has the most impact on the uptake capacity relative to the other modifiers. The magnetic materials especially magnetic biochars are in the category of carbon which can remove the pollutants by electrostatic attraction between the pollutants and oxygen-containing functional groups. Additionally, the generation of magnetic biochar at a minimal oxygen supply increases the crystalline architecture of this adsorbent owing to its graphitic domains that are much smaller in comparison with the nanocarbon materials. Magnetic materials have some benefits including: (1) They can be synthesized in one step only causes decreasing energy loss, (2) They have a flexible and exclusive property due to their surface area, high adsorption capacity, and high degree of surface reactivity, (3) They are mostly known as the economic and cost-effective adsorbents, and (4) They can reduce the concentration of the toxic gases, metallic ions, and other pollutants dramatically^25^.

The RH and modified RH were exploited as the adsorbents mostly for the removal of different dyes, and heavy metal ions. In addition, it was observed that the RH could be employed for the adsorption of some drugs such as paracetamol and tetracycline (Table 1). The use of RH and modified RH for the adsorption of AA has not been observed until now. Homagai et al. used modified RH for the removal of the crystal violet (CV) dye. The modification agent was xanthate. The highest adsorption capacity has been determined at 90.02 mg/g at a pH of 10 and a contact time of 60 min^26^. Bansal et al. used raw RH for the elimination of Cd(II) ions. The adsorption capacity was obtained at 3.83 mg/g at a pH of 2, and a time of 180 min^27^. Chowdhury et al. adsorbed malachite green using NaOH-rice husk. In their work, the uptake capacity has been obtained at 10 mg/g^28^. El-Shafey used sulfuric acid-rice husk for the removal of Se(IV) ions with an uptake capacity of 12 mg/g^29^. Hsu et al. removed the methacrylic acid using carboxyl-rice husk with an uptake capacity of 317.70 mg/g after 45 min^30^. Chen et al. could adsorb tetracycline after 40 min with an uptake capacity of 8.37 mg/g^31^. The other studies were brought in Table 1.

**Table 1:** Some works in the adsorption process using RH and modified RH.

Adsorbent	Adsorbate	T (°C)	pH	Dose (g)	C_e_ (ppm)	Time (min)	q (mg/g)	Ref.
Xanthate-rice husk	CV	25	10	0.03	1000	60	90.02	^26^
Rice husk	Cd(II)	25	2	20	100	180	3.83	^27^
NaOH-rice husk	Malachite green	25	5.50	1	100	180	10	^28^
Sulfuric acid-rice husk	Se(IV)	25	1.50	0.10	50	400	12	^29^
Carboxyl-rice husk	Methacrylic acid	37.50	7	0.20	80	45	317.70	^30^
NaOH-Rice husk	Enrofloxacin	36.43	5.11	0.69	25.02	30	241	^32^
Ca(OH)_2_-Rice husk	Pb(II)	25.30	5	0.05	600	240	350	^33^
Rice husk	Tetracycline	40	2	2	5	600	8.37	^31^
Rice husk	Paracetamol	45	7	5.50	35	90	1.31	^34^
Rice husk	Er(III)	60	3.50	0.03	300	60	250	^35^

The objective of the present work was to exploit magnetic RH for trapping vitamin C from an aqueous solution. One of the strengths of this manuscript is the adsorption of vitamin C from an aqueous solution for the first time. The novelty of this work is using agricultural waste as a cost-effective and environmentally friendly adsorbent for reducing the AA from the pharmaceutical and food industries. It will help the industries for adsorbing the AA from the effluents and afterward leaching the AA from this adsorbent and using AA again in the production cycle. The other novelty is detecting the optimum point of the effective factors for increasing the yield using design-expert software. For this aim, various effective parameters including AA concentration, the dosage of the adsorbent, and contact time have been evaluated. Besides, the optimum operation conditions are also attained using RSM. Ultimately, the reusability of the magnetic RH is evaluated for estimating its potential and capability at a larger scale.

## Material and methods

### Chemicals

In this experiment, Ascorbic acid, ferrous sulfate, and ferric chloride were utilized for the magnetization of RH. Ammonium hydroxide (25% v/v) and potassium hydroxide were used as activators. Potassium iodate, potassium iodide, and sulfuric acid were used to prepare the iodine solution. In addition, deionized water was applied as a solvent in all experiments. All used reagents were purchased from Merck Company with high purity (Table 2).

**Table 2:** The used chemical reagents in this work.

Chemical reagent	Chemical structure	Molecular weight (g/mol)	% Purity	Company	CAS number
Ascorbic acid	C_6_H_8_O_6_	176.124	> 99	Merck	50-81-7
Ferrous sulfate	FeSO_4_·7H_2_O	151.908	> 99	Merck	7720-78-7
Ferric chloride	FeCl_3_·6H_2_O	162.204	> 99	Merck	7705-08-0
Ammonium hydroxide	NH_4_OH	35.040	> 99	Merck	1336–21-6
Potassium hydroxide	KOH	56.105	> 99	Merck	1310-58-3
Potassium iodate	KIO_3_	214.001	> 99	Merck	7758-05-6
Potassium iodide	KI	166.002	> 99	Merck	7681–11-0
Sulfuric acid	H_2_SO_4_	98.079	> 99	Merck	7664-93-9

### Synthesis of modified sorbent

**Figure 1:**
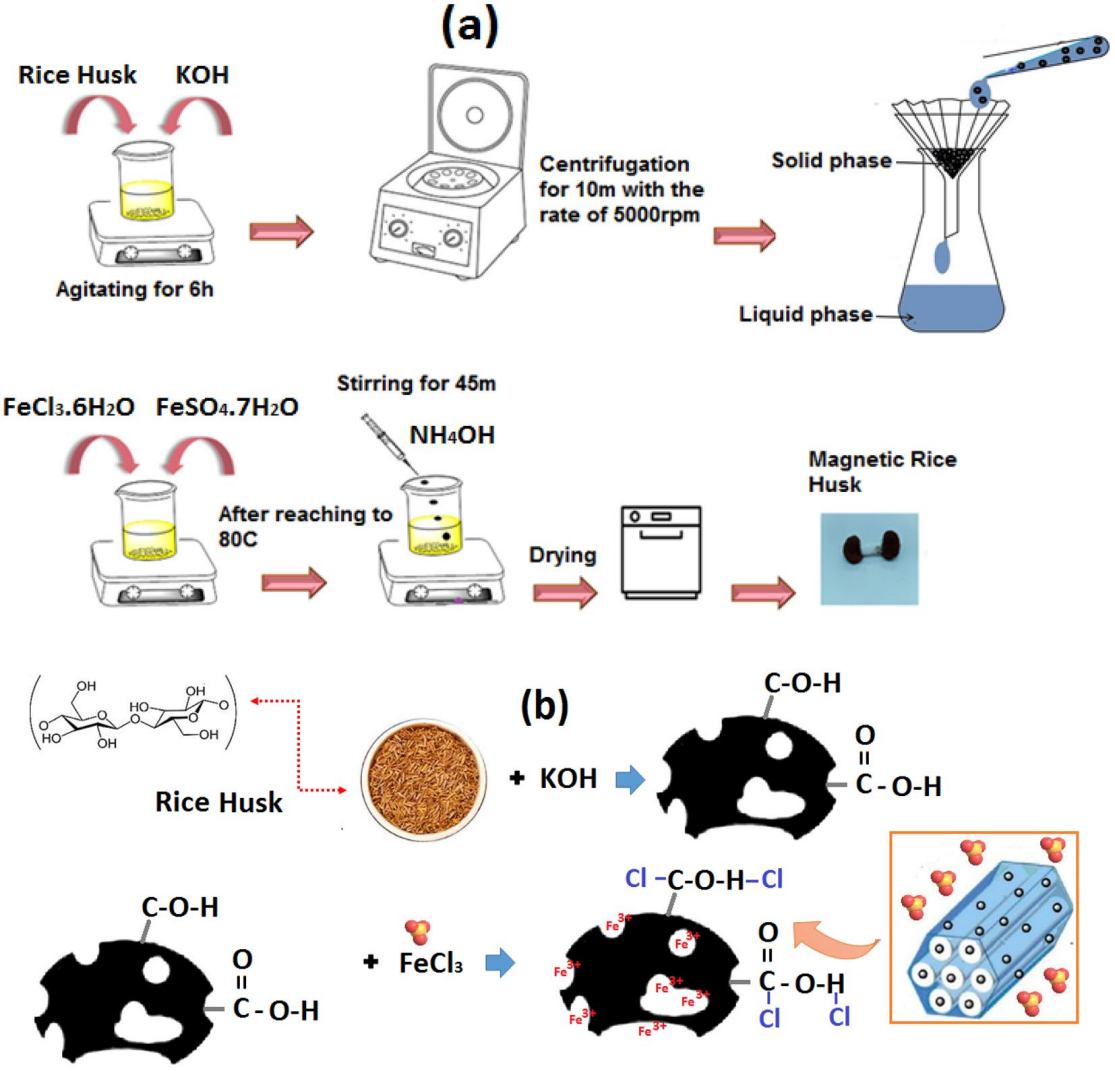
Schematic for the (**a**) synthesis of magnetic RH, and (**b**) chemical structure of synthesis process.

Rice Husk (RH) has been provided from Kozan (Shaft, Guilan, Iran) village farms. The RH has been rinsed with deionized water frequently to eliminate the dust completely. Then, it has been dehydrated in the oven at 90 °C for 24 h until it reaches constant weight, after, turn it into a micro size by the mill. The RH was modified by immersion of 20 g RH into 100 mL of KOH solution at a concentration of 1 M and then stirred for 6 h. The solid was acquired via centrifugation at the rate of 5000 rpm for 10 min. Subsequently, it has been rinsed with deionized water to reach neutral pH. The precipitation method was used to magnetize the RH-KOH. For this aim, 4.1 g of FeCl_3_ and 2.1 g of FeSO_4_ have been mixed in 80 mL of deionized water under a certain agitation rate. Afterward, 10 mL of 25% v/v of ammonia solution and a certain dose of RH-KOH were added. The magnetization reaction proceeded for 45 min at 80 °C on a stirrer at 450 rpm. Eventually, the black solid product have been attracted using magnets, rinsed several times with deionized water, and dehydrated at 60 °C for 12 h (Fig. 1). The formula of the magnetic reaction has been brought in Eq. (1).

1 $$ {\text{FeSO}}_{{4}} \cdot {\text{7H}}_{{2}} {\text{O + 2FeCl}}_{{3}} \cdot {\text{6H}}_{{2}} {\text{O + 8NH}}_{{4}} {\text{OH }} \to {\text{Fe}}_{{3}} {\text{O}}_{{4}} + {\text{6NH}}_{{4}} {\text{Cl + (NH}}_{{4}} {)}_{{2}} {\text{SO}}_{{4}} {\text{ + 17H}}_{{2}} {\text{O}} $$

### Preparation and standardization of iodine solution

**Figure 2:**
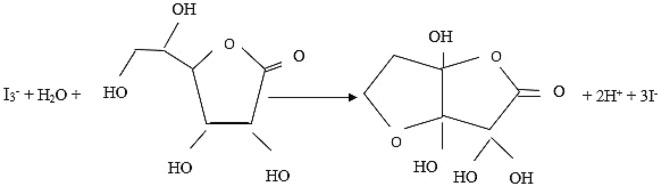
The reaction between AA and iodine ions during the titration^36,37^.

To prepare the standard iodine solution, 0.15 g of potassium iodate (KIO_3_) was dissolved in a certain amount of deionized water. Then, 3 g of potassium iodide (KI) has been added to the mixture and its volume has been increased to 100 mL. The vessel containing the materials has been mixed using a stirrer to dissolve completely. Afterward, 1 mL of H_2_SO_4_ with a concentration of 9 M was gradually introduced into the solution for accelerating the reaction until the appearance of the mixture altered from milky to dark red. Regarding the following reaction, 0.021 M of iodine solution was produced which was made fresh for daily consumption in order to obtain the desired result of the titration process. The reaction of AA with the iodine ions was displayed in Fig. 2.

### Adsorbent characterization

The participant functional groups and the variations on the adsorbent before and after the adsorption were performed using Fourier transform infrared spectrophotometer (FTIR) at the wavelength of 400–4000 cm^−1^ (AVATER, Thermo, USA). The morphological image and compositions of the adsorbent (SEM–EDX) were determined by scanning electron microscopy (Mira3, Tescan, Czech Republic). Fe_3_O_4_ composition was characterized by X-ray fluorescence using a diffractometer (Pw1730, Philips, Netherlands) with Cu/Kα radiation (40 kV, 30 mA). Also, a vibrating sample magnetometer (VSM) model of MDKB 4214 was employed for measuring the magnetic property of modified RH. The specific surface area has been identified by applying the Brunauer–Emmett–Teller (BET) equation. The heat stability of the synthesized adsorbents has been investigated by applying the thermogravimetric test (TGA, Model 2960, Universal V2.4F TA Instruments, USA).

### Adsorption

The adsorption experiments have been accomplished in the beaker containing 50 mL of AA solution and modified RH. All experiments were performed at room temperature and stirred at 450 rpm, The adsorbent dosage, AA concentrations, temperatures, and contact time were selected in the domain of 0.5–1 g, 400–800 mg/L, 288–308 K, and 10–120 min for finding the best performance conditions of the resin, respectively. After the adsorption process, the resulting suspension was placed in a centrifuge at 3000 rpm for 10 min. The content of the adsorbed AA has been measured using titration with iodine solution in the presence of starch as an indicator. The adsorption process has been repeated in three epochs to reduce the error of the experiment and their mean values were reported. The uptake capacity at the saturation state (qe) and removal efficiency of AA were calculated by the following formulas:

2 $$ {\text{q}}_{{\text{e}}} = \frac{{{\text{(C}}_{{0}} - {\text{C}}_{{\text{e}}} {\text{)V}}}}{{\text{W}}} $$

3 $$ {\text{\% R}} = \frac{{{\text{(C}}_{{0}} - {\text{C}}_{{\text{e}}} {)}}}{{{\text{C}}_{{0}} }} \times { 1}00\% $$

4 $$ {\text{R}}_{{\text{L}}} = \frac{{1}}{{{1} + {\text{K}}_{{\text{L}}} {\text{C}}_{{0}} }} $$

where C_0_ is the initial concentration of vitamin C (mg/L); C_e_ is the equilibrium concentration of vitamin C (mg/L); V is the volume of vitamin C solution (L); and W is the dosage of modified RH (g).

### Experimental design

Response surface methodology (RSM) is the kind of statistical procedure with the basis of math to obtain the best-predicted models. The aim of this program is to optimize the dependent variable with the help of polynomial equations, which are impacted by independent factors. This technique is popular because it reduces the number of tests, reduces cost and time, and has good precision^38^. Recently, the use of RSM as a useful method has been expanded compared to the classical method because, in the classical test, the effect of the interaction of the factors on the response is not examined, which causes an error as a result of the experiment. For instance, Chowdhury et al. in 2013 used RSM for optimizing the adsorption of crystal violate with the help of NaOH-modified rice husk. They used design-expert software version 7.1.6. The factors of dye concentration (100–200 mg/L), flow rate (10–30 mL/min), pH, and bed height (5–25 cm). The optimized conditions have obtained pH of 8, concentration of 100 mg/L, flow rate of 22.88 mL/min, and bed height of 18.75 cm^39^. Popoola in 2019 used a 2-level factorial design for optimizing the experimental conditions such as temperature (600–1000 °C), time (1–5 h), mixing ratio (1–5), and magnetite loading (2–10 wt%) by helping design-expert version 7.0.7. The optimum values of temperature, time, mixing ratio, and magnetite loading were predicted at 859.20 °C, 2.32 h, 2.54, and 5.56 wt%^40^. In this research, the central composite design (CCD)^41^ with four autonomic parameters (m = 3) such as acid concentration (X_C0_ = 400–800 ppm), contact time (X_t_ = 10–120 min), adsorbent dosage (X_D_ = 0.5–1 g) has been applied for inspecting the separation of AA from the liquid phase. In this experiment, all 3 factors were designed and coded at 5 levels, which are as follows: (− 2, + 2) the lowest and highest level, (− 1, + 1) low and high level, and 0 central points, respectively. Also, The total number of experiments was ordained 20 = 2 m + 2 m + 6 that 16, 8, and 6 are factorial points, axial points (star), and central points, respectively^42^. The relevance between the response and the factors examined was entailed by a quadratic equation:

5 $$ \begin{aligned} Y \, = & A_{0} + \, A_{1} X_{co} + \, A_{11} X_{co}^{2} + \, A_{2} X_{t} + \, A_{22} X_{t}^{2} + \, A_{3} X_{D} + \, A_{33} X_{D}^{2} + \, A_{12} X_{co} X_{t} + \, A_{13} X_{co} X_{D} \\ & + \, A_{23} X_{t} X_{D} + \varepsilon \\ \end{aligned} $$

Y illustrates the percentage of AA adsorption (predicted response); X_c0_, X_t_, and X_D_ are autocratic coded factors in the experiment. Furthermore, A_0_, A_i_, A_ii_, and A_ij_ refer to the coefficients of the linear, quadratic, and interaction between them, respectively, and are random inaccuracy. The significance of the expanded regression models was estimated through the analysis of variance (ANOVA). The design of the experiment for the present adsorption process has been examined using the central composite design (CCD) method under RSM to determine the optimal conditions.

According to Table 3, we define three symbols in the design-expert software such as A, B, and C, which refer to the acid concentration, adsorbent dosage, and contact time, respectively. Also, the value of α was defined at a number of 2 in this software, thus, the central point of the acid concentration, adsorbent dosage, and contact time was calculated at 600, 0.75, and 70, respectively. As can be seen, the number of the levels for these three parameters is 5. The ranges of the experimental parameters were selected with respect to the Refs.^43–45^.

**Table 3:** The levels of the effective parameters with the α of 2.

Parameters	Symbol	α −	+ α	0
Concentration (ppm)	A	400	800	600
Adsorbent dosage (g)	B	0.5	1	0.75
Contact time (min)	C	10	130	70

## Results and discussion

### Characterization analysis

**Figure 3:**
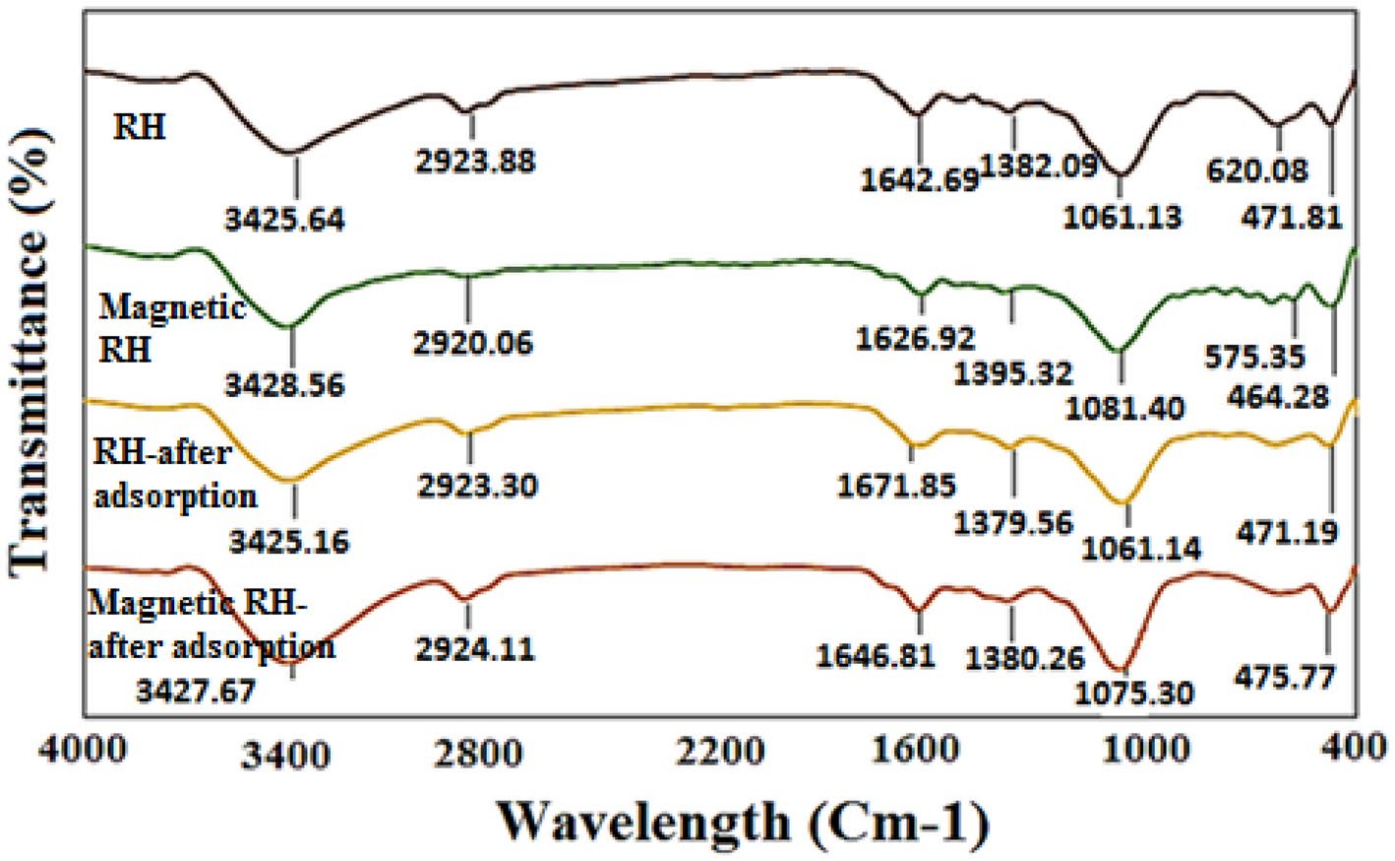
The FTIR image of RH, Magnetic RH, Adsorbed AA on the RH, and Adsorbed AA on the Magnetic RH.

Figure 3 displays the FTIR of the adsorbents for determining the wavelength of the functional moieties in the adsorbent before and after AA adsorption. The peak in the wavelength of 3427 cm^−1^ was ascribed to the water molecules and the tensile vibrations of the hydroxyl group (–OH) which was due to the intermolecular hydrogen bonding of some reagents like alcohols, phenols, and carboxylic acids in the lignin and cellulose structure. Hydroxyl moieties are on the adsorbent plane. The peak in the wavelength of 2929 cm^−1^ is attributed to the symmetric and asymmetric tensile vibrations (C–H) of aliphatic acids in cellulose (which is present in the RH structure), which indicates the presence of an alkane functional group (–CH_3_ or –CH_2_)^46^. The peaks in the wavelength of 11,646 cm^−1^ correspond to asymmetric mobility of (C=O) (carbonyl groups (ketones and aldehydes), carboxylic acid or ester, as well as to tensile vibrations (CN) and flexural vibrations (NH) related to proteins^47^. The peak is also noted at the wavelengths of 1075 cm^−1^, and 1646 cm^−1^, which are attributed to the Si–O–Si tensile vibrations and the asymmetric tensile vibrations of C=O, respectively. Fe–O bonds are vibrating on the surface of the material at the wavelength of 400–600 cm^−1^^48,49^. As can be seen, after modifying the RH with magnetic iron nanoparticles, a peak with a wavelength of 575–135 cm^−1^ is formed, which is owing to the mobility of Fe^3+^ and O^2-^ in the structure of Fe_3_O_4_^50,51^. Comparison of Fourier transform infrared spectroscopy before and after adsorption of AA shows that the peaks in the wavelength range of 3427, 2924, and 1646 cm^−1^ are ascribed to the hydroxyl functional moiety, symmetric and asymmetric tensile vibrations of C–H, and asymmetric (C=O), respectively. The peak is weakened in the wavelength of 1075 cm^−1^ which is attributed to the Si–O–Si tensile vibrations, which indicates the involvement of these factor groups during the adsorption process. In addition, the peak of Fe–O is amplified after the adsorption process, which can be analyzed in such a way that magnetic iron nanoparticles have participated in the adsorption procedure^19,20,52^.

**Figure 4:**
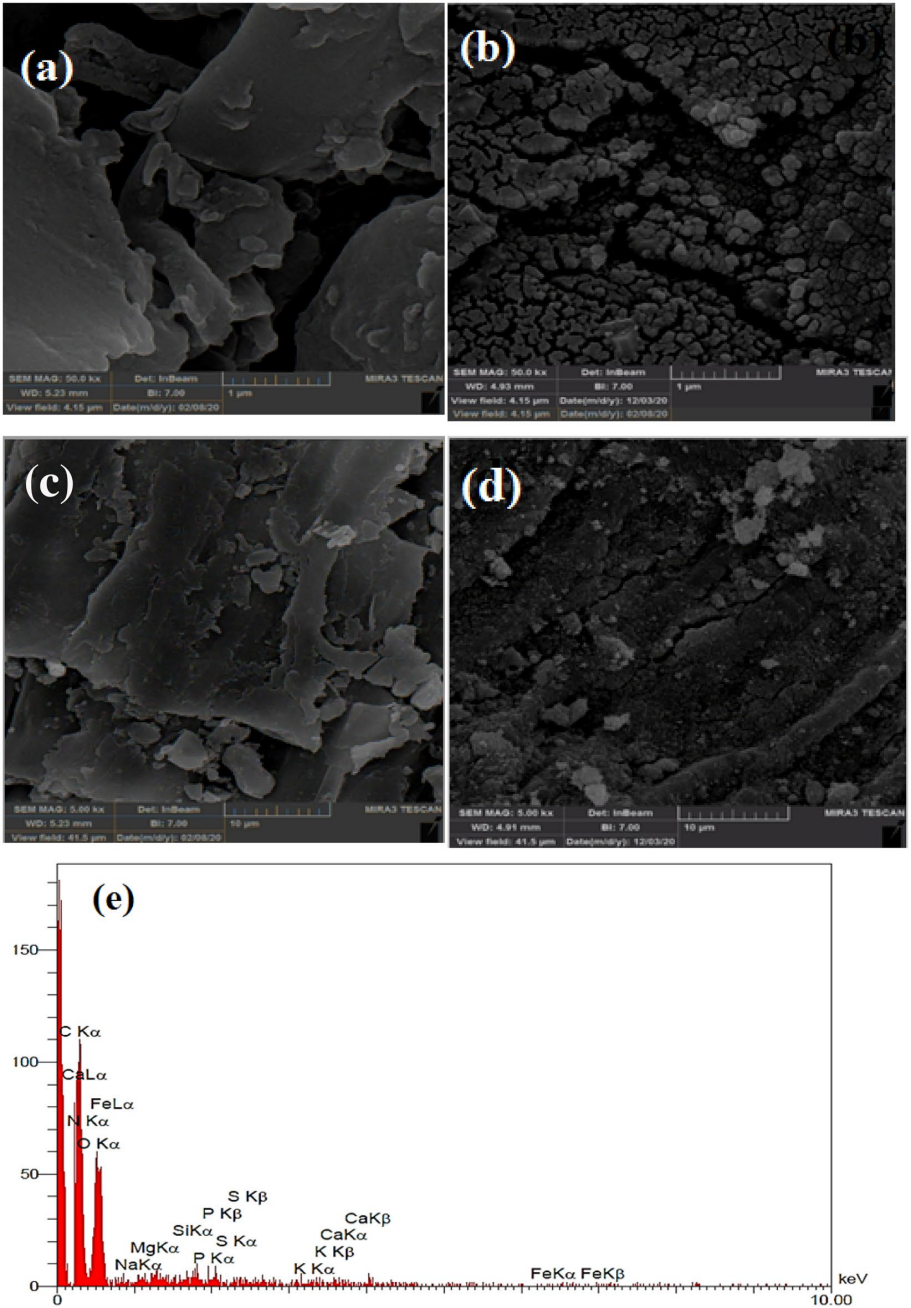

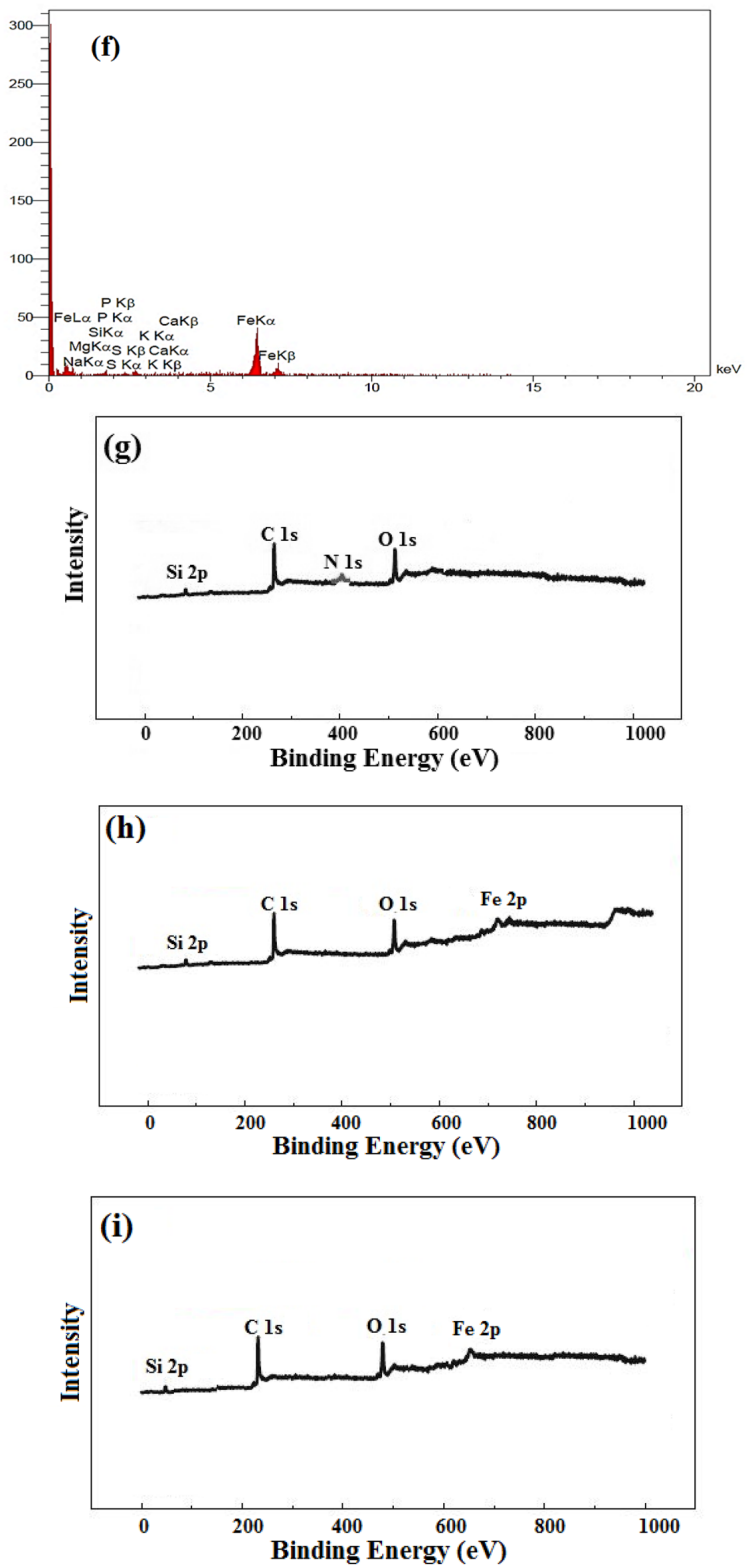
The SEM image of (**a**) RH, (**b**) Magnetic RH, (**c**) Adsorbed AA on the RH, and (**d**) Adsorbed AA on the magnetic RH. The EDX image of (**e**) RH, and (**f**) Modified RH. The XPS image of (**g**) raw, and (**h**) magnetic RH.

SEM test has been performed for studying the morphology of the RH surface before and after AA uptake. The SEM micrograph of the RH shows that the surface has a smooth surface (Fig. 4a) and after the modification, many pores have been dispersed through the RH and its porosity has increased (Fig. 4b). Figure 4c and d also showed the structure of the RH and magnetic RH after the adsorption of AA, respectively. Because of the accumulation of AA molecules, the adsorbent surface became smoother and had fewer depressions, which generally indicates the successful adsorption of AA on the adsorbent surface. The EDX patterns for the detailed study of compounds present in the RH and magnetic RH are displayed in Fig. 4e and f, respectively. This analysis confirms the presence of the iron particles after the modification. The EDX test also implied that the RH contains very small amounts of minerals and carbohydrates such as cellulose and lignin in its structure. Thus, the main elements in the structure of the RH are oxygen, carbon, and a small amount of iron^53^. Also, Fig. 4f is related to magnetic RH, which, as can be seen from the composition of the percentage, after placing the iron nanoparticles on the RH, the percentage of iron's composition has increased significantly and their carbon and oxygen have diminished, because the nanoparticles are well deposited on the surface of carbon, which is the main constituent of bran^54^. The main constituents of RH are about 70% of the inorganic part of rice bran, and it is mainly composed of silica and small amounts of alkali metal oxide. RH is composed mainly of lignin (20–30%), holo-cellulose (55–65%), SiO_2_ (15–20%) and extracts (2–5%), which can be regarded as a natural organic–inorganic composite (Table 4).

**Table 4:** The compositions percentage in the RH.

Component	Percentage
Cellulose	32.34
Hemicellulose	21.62
Lignin	21.55
Inorganic components	15.14
Water	8.06
Other things	0.28

The XPS (X-ray photoelectron spectroscopy) images are illustrated in Fig. 4. The model of the XPS apparatus is SPECS FlexMod. Figure 4g and h show the XPS image of the raw and magnetic RH. In addition, the XPS image after the adsorption is depicted in Fig. 4i. The peaks in Fig. 4g are 285, 533, 400, and 103.50 eV, which relate to the C1*s*, O1*s*, N1*s*, and Si2*p*, respectively. The peak at the binding energy of 750 eV in Fig. 4h appears which corresponds to the Fe2*p* because of the presence of the iron elements in the magnetic RH. Figure 4h implies that the magnetization has occurred successfully. According to Fig. 4i, the binding energy of peaks for C1*s*, O1*s*, Si2*p*, and Fe2*p* has declined which is owing to the chelation of AA to these atoms, and it reveals the successful adsorption of AA onto the magnetic RH. The binding energy of C, O, Si, and Fe after the adsorption of AA are 250, 500, 400, and 95 eV, respectively^52^.

**Figure 5:**
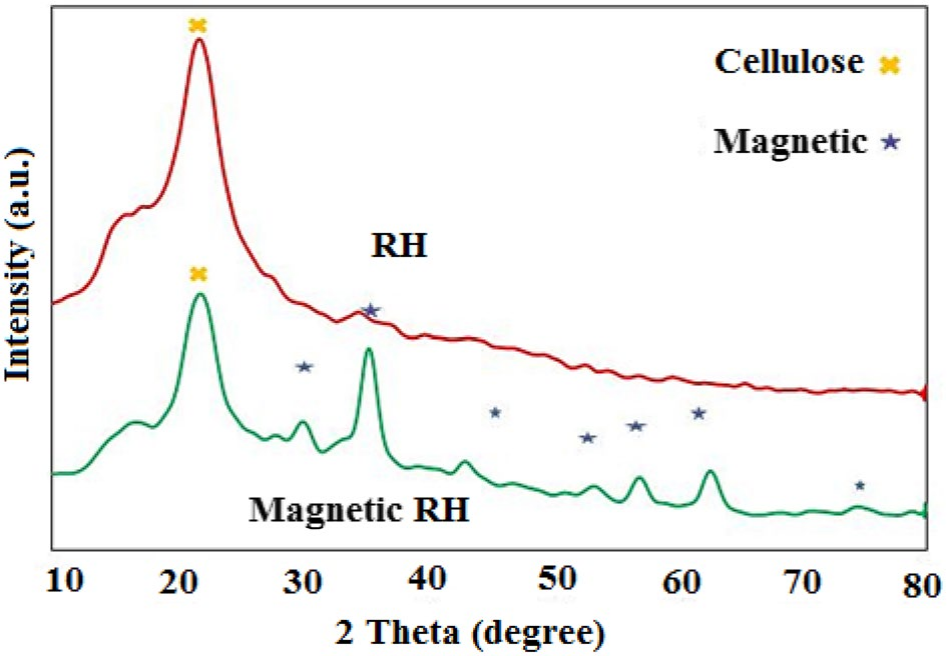
The XRD diagram of RH and magnetic RH.

The X-ray diffraction patterns (Card No. [75-0033 Joint Committee on Magnetic Diffraction Standard (JCPDS)]) for RH and M-KOH-RH adsorbents have been shown in Fig. 5. According to Fig. 5, it is obvious that RH has not a crystalline structure, because of a sharper peak at 2θ of 22.19°, which is ascribed to the existence of organic reagents in the structure of RH (cellulose, hemicellulose, and lignin) and prove the lack of mineral compounds. XRD pattern also reveals the amorphous structure of the RH. The diffraction patterns of Fe_3_O_4_ in the modified RH are 30.34, 36.39, 49.43, 53.59, 34.57, 94.62, and 54.74° indicating the presence of magnetic particles on the surface of the RH^55,56^.

**Figure 6:**
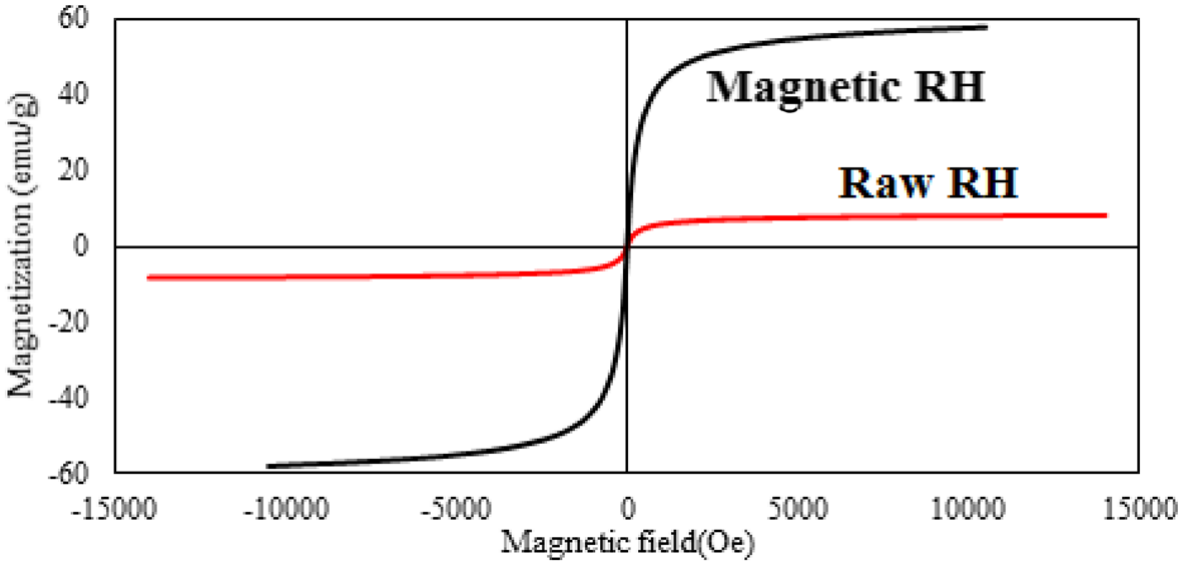
The VSM diagram of the raw (red line) and magnetic RH (black line).

One of the disadvantages of traditional adsorbents is their separation from the solution, which prevents the adsorbent from reusing. The addition of iron not only increases the adsorption capacity but it can also be conveniently isolated from the adsorbent via employing a magnetic field. Figure 6 shows the magnetic properties of raw and modified RH with a vibrating sample magnetometer (VSM) system. The magnetic field was adjusted in the domain of − 15,000 to + 15,000 Oe at room temperature. Saturation magnetization values for the raw and magnetic RH have been determined at 8 and 57 emu/g, respectively. Regarding Fig. 6, the magnetic property of the raw RH is less than that of the magnetic one, which means that the Fe_3_O_4_ particles have occupied the structure of rice successfully.

**Figure 7:**
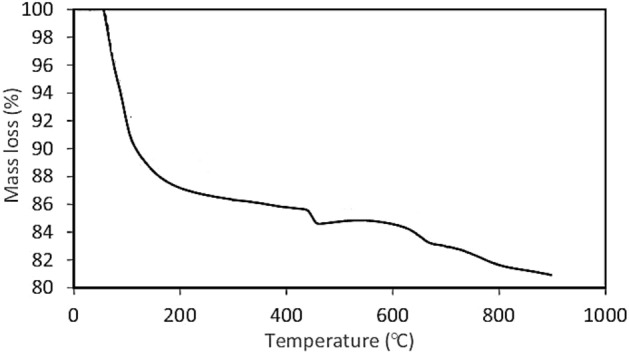
The TGA diagram of the magnetic RH.

The BET analysis for the RH and modified RH was conducted. The surface area of the RH and modified RH was determined at 98.17 and 120.23 m^2^/g, respectively. Besides, the pore volumes of the RH and modified RH have been obtained at 2.10 and 6.20 mm^3^/g, respectively. It reveals that the modification of RH increases the surface area, which causes improving the adsorption of AA onto the RH due to the increment of pores on the RH. Besides, the TGA analysis of the modified rice husk was performed and it is illustrated in Fig. 7. Regarding this figure, the thermal stability of the modified rice husk was 450℃, which demonstrates the high resistance of the modified rice husk to the temperature elevation.

### RSM results

Variance analysis for the adsorption efficiency of AA is conducted by considering the temperature, acid concentration, and dosage in Table 6. In fact, the existence of P-value, F-value, and lack of fit can help us to decide about the significance or insignificance of these parameters. The other case that was brought in this section is predicting an equation for the adsorption process. The equation in terms of actual factors can be used to make predictions about the response for given levels of each factor, thus, defining an appropriate model or equation for the process can help us to describe the behavior of the system better. According to Table 5, different polynomials such as linear, quadratic, and cubic were fitted versus the experimental data in the design-expert software for selecting the closest model to the empirical results. This table reveals that the quadratic polynomial was suggested, thus, the experimental parameters have the degree of two. Equation (6) was also predicted by the software, which explains that there is an optimum point at the center of the quadratic graph instead of the corner points. Besides, this equation should not be used to determine the relative impact of each factor because the coefficients are scaled to accommodate the units of each factor and the intercept is not at the center of the design space. Regarding Table 6, the Predicted R^2^ of 0.9488 is in reasonable agreement with the Adjusted R^2^ of 0.9860. Adeq Precision measures the signal-to- noise ratio. A ratio greater than 4 is desirable. The calculated Adeq Precision is 47.553 which indicates an adequate signal. This model can be used to navigate the design space. Regarding Table 7, the P-value of the model has attained lesser than 0.0001 and demonstrated that the models are significant. The F-value for the adsorption percentage is measured at 102.15, which proved the models are significant. There is only a 0.01% possibility that F-values these large could occur because of the noise ^57^. Regarding Table 6, each model hasn’t lack fit, which proved that the model is appropriate. Regarding Table 6, the P-values of acid concentration, adsorbent dosage, and time have been obtained lesser than 0.0001, which shows the effectiveness of these factors. Table 6 also proves that the interaction between the acid concentration with time, acid concentration with adsorbent dosage, and adsorbent dosage with time are effective on the adsorption efficiency because their P-values are attained at 0.0075, 0.0048, and 0.0064 which are lesser than 0.05^58^. The model F-value for response is determined 102.15, which indicated the model is significant. There is only 0.01% probability that F-value this large could happen own to noise. In addition, the degree two of the time is effective because its value is 0.0024. According to the RSM results, the optimum experimental conditions have been predicted at the acid concentration of 486.929 ppm, adsorbent dosage of 0.875 g, and time of 105.397 min. In addition, the adsorption efficiency has been obtained at 92.936% at these optimum conditions with the desirability value of 1.00 which is the best value. It is essential to refer to this notice that the values of the acid concentration, adsorbent dosage, and contact time were selected “in range” and the value of response or % R was selected “maximize” in the design-expert software, because our goal was maximizing the adsorption efficiency with respect to the range values of the experimental conditions (Table 8). In Eq. (6), the symbols A, B, and C are ascorbic acid concentration, adsorbent dosage, and contact time, respectively. We have obtained Table 8 by using experiment design software. In this way, we selected the investigated parameters which include the initial concentration, adsorbent dose and contact time by selecting the in range option in the desired range. The purpose of this work is to show values of parameters where R has its maximum value. For this reason, we choose the highest option for R, which is the absorption percentage value, so that the software is able to protect the optimal R value. As shown in the table, in the initial concentration = 718.92 ppm, adsorbent dose = 0.898 mg and contact time = 105 min, we reach the maximum value of absorption percentage which is 92.33%. Also, in the initial concentration = 481 ppm, adsorbent dose = 0.601 mg and contact time = 34 min, we reach the lowest absorption percentage value, which is 25%.

6 $$ \begin{aligned} {\text{\% R}} = & + {33}{\text{.36}} - {0}{\text{.11A}} + {99}{\text{.96B}} + {0}{\text{.41C}} - {0}{\text{.037AB}} - {0}{\text{.0000178AC}} + {0}{\text{.000065A}}^{{2}} \\ & - \;{49}{\text{.37B}}^{{2}} - {0}{\text{.001985C}}^{{2}} \\ \end{aligned} $$

**Table 5:** Comparison predicted models.

Source	Sum of squares	df	Mean square	F-value	P-value	
Mean vs total	69,683.73	1	69,683.73			
Linear vs mean	5481.49	3	1827.16	115.57	< 0.0001	
2FI vs linear	85.52	3	28.51	2.21	0.1353	
Quadratic vs 2FI	125.16	3	41.72	9.87	0.0025	Suggested
Cubic vs quadratic	28.51	4	7.13	3.10	0.1046	Aliased
Residual	13.78	6	2.30			
Total	75,418.19	20	3770.91

**Table 6:** The statistical results for the experimental and predicted results.

Std. dev	2.06	R^2^	0.9926
Mean	59.03	Adjusted R^2^	0.9860
C.V. %	3.48	Predicted R^2^	0.9488
		Adeq Precision	47.5527

**Table 7:** The ANOVA results for the adsorption of AA on the modified RH.

Source	Squares sum of	df	Square mean	F-value	P-value
Model	5557.14	9	617.46	102.15	> 0.0001
A-Concentration	1113.18	1	1113.18	184.16	> 0.0001
B-Sorbent	588.90	1	588.90	97.43	> 0.0001
C-Time	3649.34	1	3649.34	603.75	> 0.0001
AB	0.64	1	0.64	0.11	0.007519
AC	3.18	1	3.18	0.53	0.004852
BC	71.40	1	71.40	11.81	0.0064
A^2^	10.25	1	10.25	1.70	0.2221
B^2^	19.73	1	19.73	3.26	0.1009
C^2^	97.84	1	97.84	16.19	0.0024
Residual	60.44	10	6.04		
Lack of fit	50.94	5	10.19		0.0445
Pure error	9.50	5	1.90	5.36	
Cor total	5617.59	19

**Table 8:** The constraints of optimization in the design of experiment.

Name	Goal	Lower limit	Upper limit	Lower weight	Upper weight	Importance
A: acid con	is in range	481.079	718.921	1	1	3
B: sorbent	is in range	0.601349	0.898651	1	1	3
C: time	is in range	34.3238	105.676	1	1	3
R	maximize	25	92.33	1	1	5

**Figure 8:**
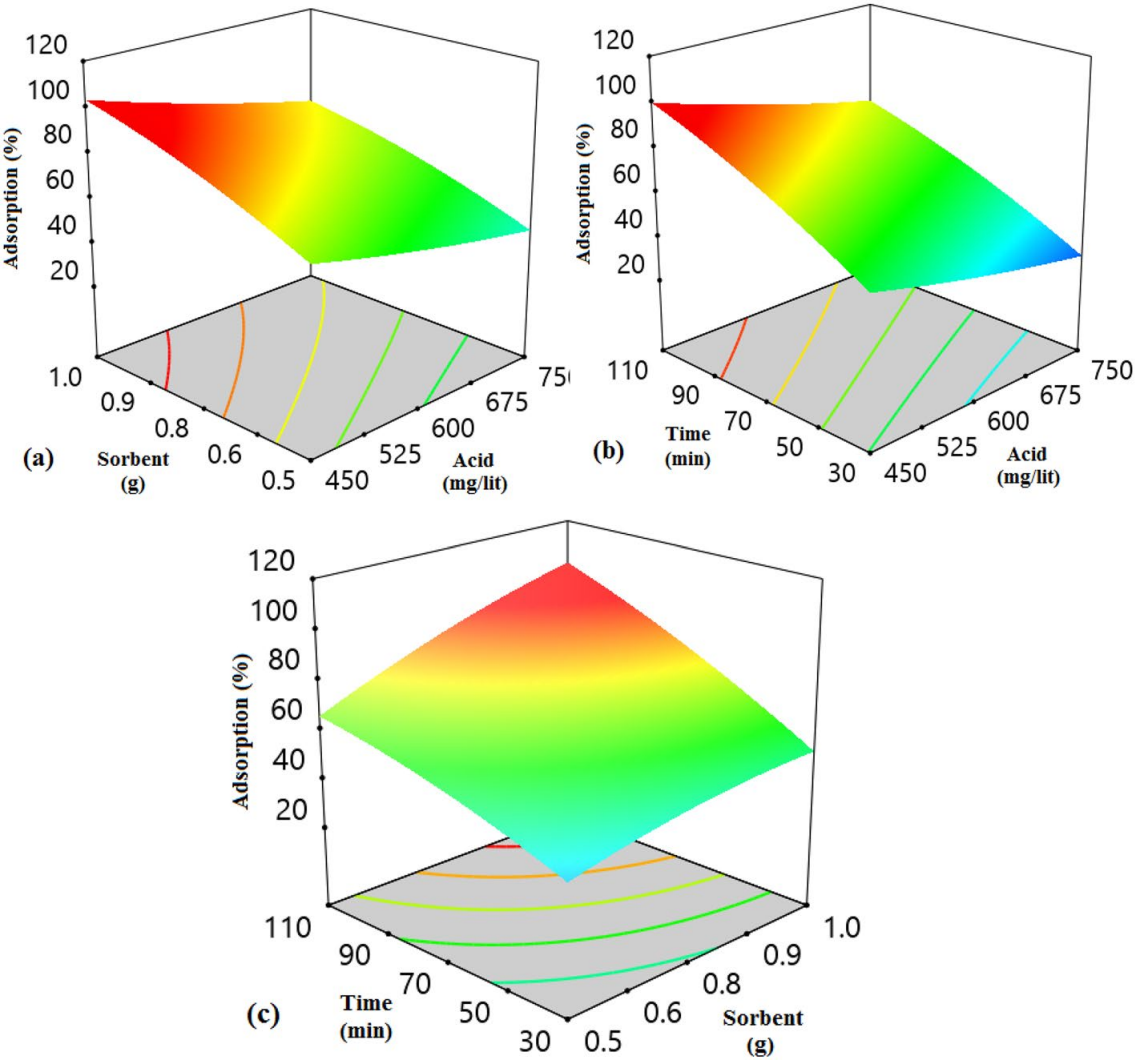
The 3D diagram for the effect of the (**a**) adsorbent dosage and acid concentration, (**b**) time and acid concentration, and (**c**) time and adsorbent dosage.

**Figure 9:**
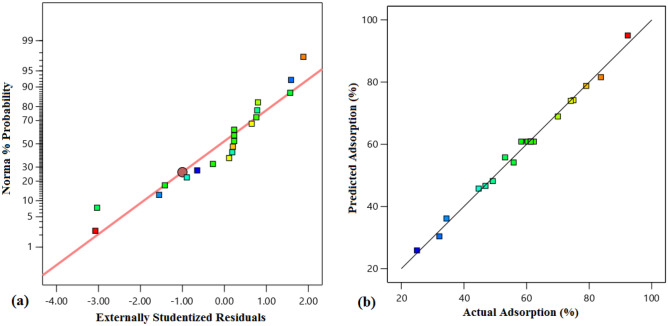
The diagram of (**a**) % probability vs. externally studentized residuals, and (**b**) predicted vs. actual values.

**Figure 10:**
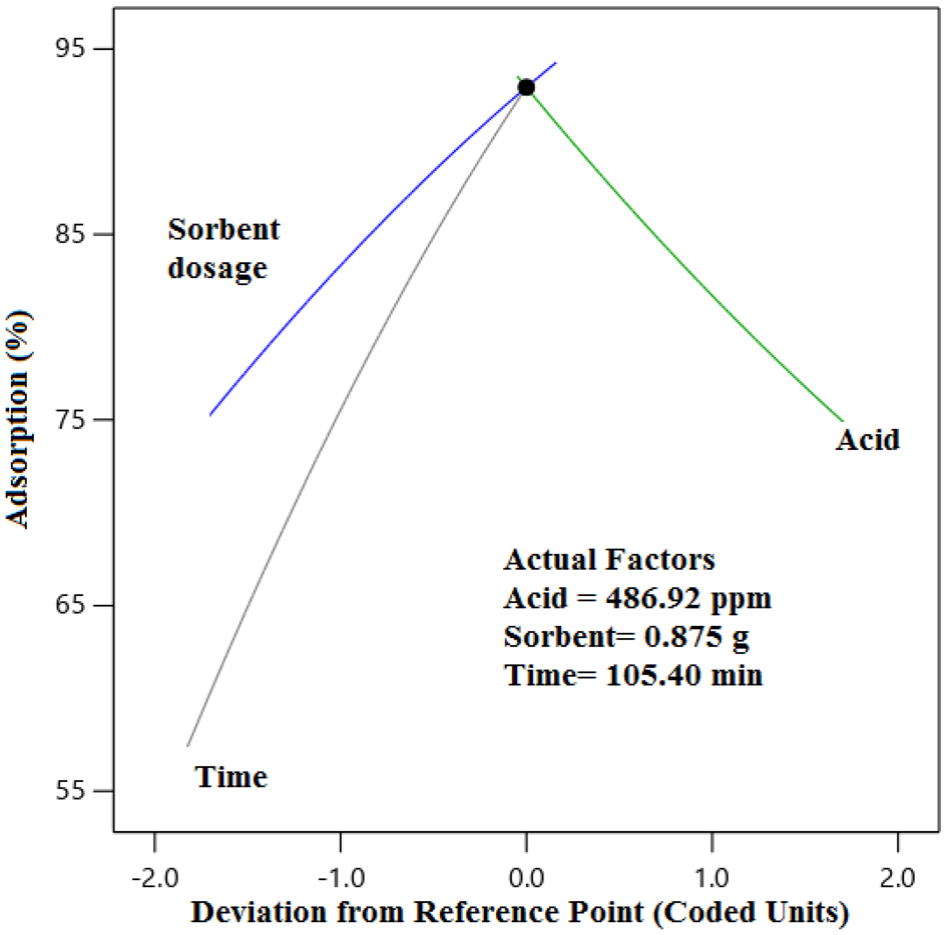
Perturbation graph of the influential parameters.

The 3D diagrams induce important information about the impact of three factors on the adsorption efficiency (response) simultaneously by analyzing the effect of interaction between two variables by setting a factor at its central level, which is displayed in Fig. 8. The observations revealed that the interaction of acid concentration and adsorbent dosage on the response was weak (Fig. 8a). As shown in Fig. 8a, the adsorption efficiency dropped mildly with increasing acid concentration, because the number of the penetrated molecules into the active centers increases and can block these centers and there is not an opportunity for the other molecule for filing the vacant cavities. Increasing the adsorbent dosage has a positive impact on the adsorption efficiency, because the number of active sites has been developed and porosity is increased (Fig. 8b,c). Figure 8b and c reveal that the adsorption occurs rapidly at the initial times, because of the presence of more unsaturated active centers at the primary times, and these sites are occupied by the AA with passing time which enhances the mass transfer resistance and the diffusion of the AA molecules is prohibited^59,60^. The normal probability graph can be utilized to control and survey the normality of the empirical data. The proximity of the squares to the direct line demonstrates the normal dispersion of the error with a mean of zero and a fixed value. According to Fig. 9a, the results of the adsorption efficiency are in the vicinity of the direct line, which reveals the normality of the obtained data of the response. The squares with various colors and straight routes are the experimental and anticipated values, respectively. The more focused the squares are on the straight route proves the better the data distribution. Regarding Fig. 9b, the adsorption percentage of AA placed close to the route displayed that the actual results obeyed a specified function, the data distribution trend was desired and the confidence in the obtained data is high^61^. The perturbation diagram assists to contrast the influence of all factors at a determined place in the design space. The responses are drawn by varying only one parameter over its limited area while keeping the other parameters unchanged. By default, Design-Expert imagines the criteria placed at the midpoint (coded 0) of the entire parameters. Figure 10 indicated that the change in the adsorption efficiency with each of the effective factors is not a direct route, which proves that the adsorption efficiency of AA is susceptible to variations of entire influential parameters in the adsorption process. Besides, each of the factors has a dissociated point where the response is enlarged. Also, it was derived from Fig. 10, increasing the adsorbent dosage and temperature and decreasing the acid concentrations from the optimized value has a suitable influence on the adsorption process and ultimately the economy of the process^61^.

**Figure 11:**
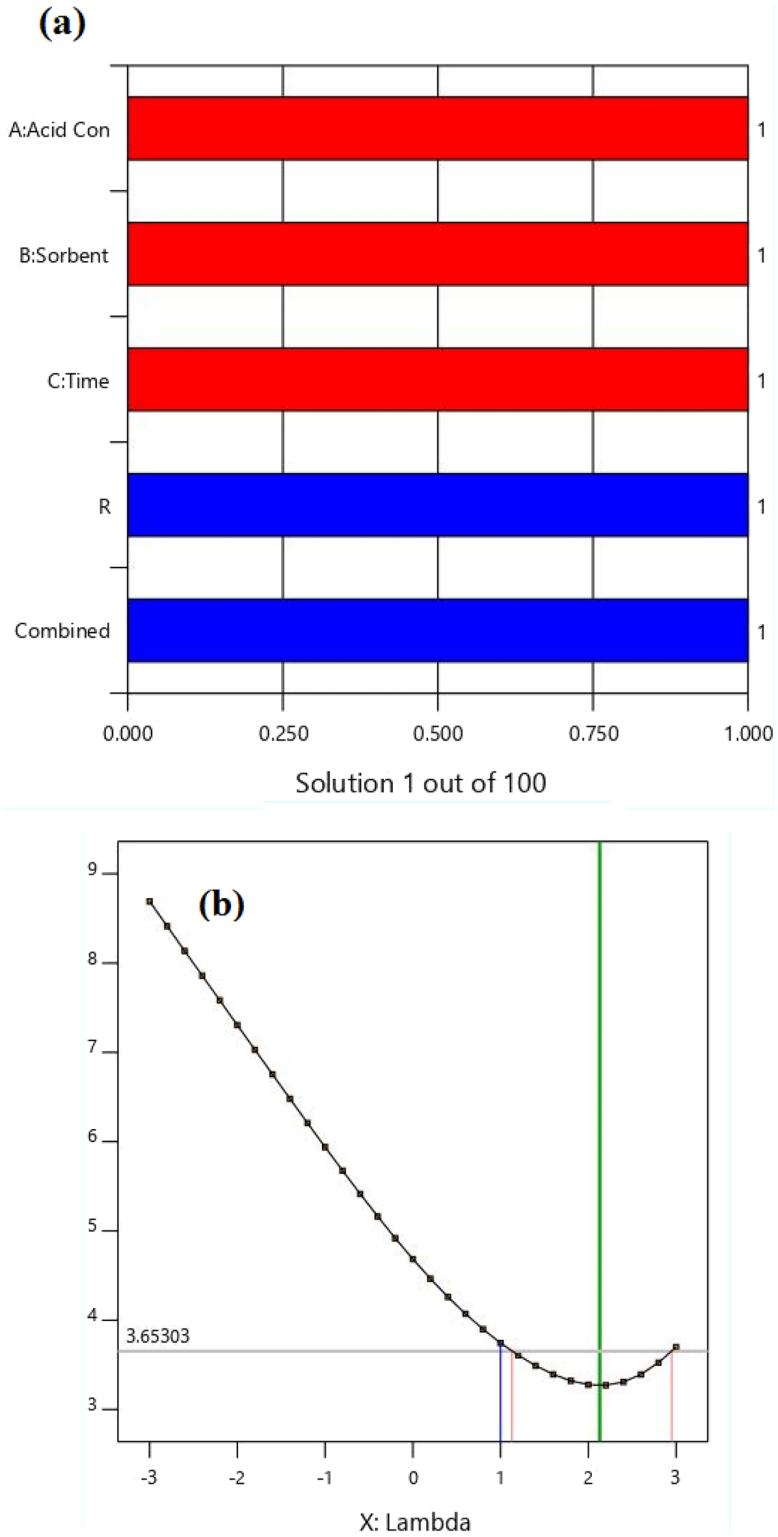
The (**a**) Pareto, and (**b**) Box–Cox diagrams.

The Box-Cox diagram set the best lambda value for the adsorption process at 2.13, indicating that the data did not need to be modified for further improvement (Fig. 11b). The Pareto diagram is another tool to investigate the significance of independent variables on a response (Fig. 11a). The pareto graph shows how well each variable satisfied the criteria: values near one are good. It is observed that the response is one and demonstrating that the aim of maximizing the adsorption percentage is occurred appropriately^20,62–64^.

### Isotherm modeling

**Figure 12:**
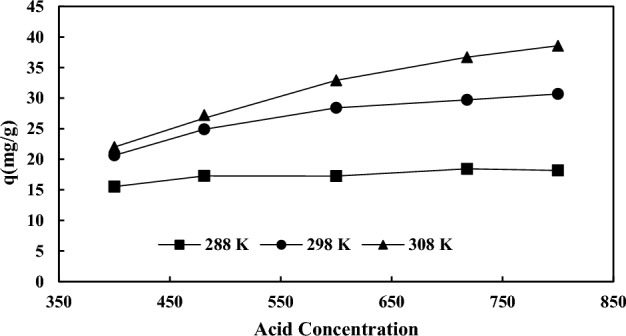
The diagram of uptake capacity vs. the acid concentration.

Isotherm study provides important information for scaling up and designing the adsorption process^65,66^. Adsorption equilibrium studies provide comprehensive information to describe the adsorption of solutes between the sorbent surface and solution, especially at the equilibrium state. Figure 12 reveals the diagram of uptake capacity against the acid concentration. Regarding this figure, the uptake capacity increases by acid concentration in temperatures of 15, 25, and 35 °C. Table 9 shows the factors of the isotherm models for the adsorption of AA on the modified KOH rice husk (M-KOH-RH) at temperatures of 15, 25, and 35℃. In the present work, the empirical results have been fitted with the four isotherm models such as Langmuir, Freundlich, Temkin, and Dubinin–Radushkevich (D–R)^67^. The equations of four isotherm models are presented below:

7 $$ {\text{Langmuir}}\;{\text{model}}:\;\frac{{{\text{C}}_{{\text{e}}} }}{{{\text{q}}_{{\text{e}}} }} = \frac{{{\text{C}}_{{\text{e}}} }}{{{\text{q}}_{{\text{m}}} }} + \frac{{1}}{{{\text{K}}_{{\text{L}}} {\text{. q}}_{{\text{m}}} }} $$

8 $$ {\text{Freundlich}}\;{\text{model}}:\;{\text{logq}}_{{\text{e}}} = {\text{logK}}_{{\text{F}}} + \left( {\frac{{1}}{{\text{n}}} \times {\text{logC}}_{{\text{e}}} } \right) $$

9 $$ {\text{Temkin}}\;{\text{model}}:\;{\text{q}}_{{\text{e}}} = \beta \, \ln \left( {{\text{A}}_{{\text{T}}} } \right) + \beta \, \ln \left( {{\text{C}}_{{\text{e}}} } \right) $$

10 $$ {\text{D}}{-}{\text{R}}: \;\ln \left( {{\text{q}}_{{\text{e}}} } \right) = \ln \left( {{\text{q}}_{{\text{D}}} } \right) - {\text{K}}\varepsilon^{2} $$

**Table 9:** The isotherm constants of AA on the M-KOH-RH.

Model	Parameter	Temperature (°C)		
		15	25	35
Langmuir	q_m,cal_ (mg/g)	19.157	31.34	38.75
	K_L_ (L/mg)	0.0396	0.0797	0.6218
	R^2^	0.9982	0.9996	0.9985
Freundlich	R_L_	0.0498	0.0254	0.0033
	K_F_ (mg/g)	9.754	13.295	24.4005
	n	9.727	6.622	10.384
	R^2^	0.815	0.9168	0.8966
Temkin	β (J/mol)	1.7377	3.8101	3.4455
	A_T_ (L/g)	81.247	11.658	623.0956
	R^2^	0.8229	0.9379	0.908
D-R	q_D_ (mg/g)	18.25	29.07	38.67
	K (mol^2^/J^2^)	0.0004	0.00005	0.00002
	E (kJ/mol)	0.0353	0.1000	0.1580
	R^2^	0.9001	0.9516	0.9959

The constants in the Langmuir, Freundlich, and Tamkin relations are K_L_, K_F_, and β, respectively. In these equations, q_e_ (mg/g), q_m_ (mg/g), and C_0_ (mg/L) refer to the saturation uptake capability, maximum adsorbent capacity, and initial adsorbent content. Also, ε is the polarization potential of the D–R equation, the constant K refers to the average free adsorption energy, and the q_D_ (mg/g) refers to the theoretical saturation absorption capability. n is also the heterogeneity coefficient of the Freundlich model. The n parameter for the AA adsorption process using modified rice husk is 10.384 which shows that the adsorption process by these adsorbents is physical and is done easily^65^. A_T_ (L/min) is an equilibrium connection constant of the Temkin model. Table 9 shows the different parameters of the adsorption isotherm with the linear fitting of four models at temperatures of 15 °C, 25 °C, and 35 °C. By comparing the regression values, it can be concluded that the Langmuir and D–R isotherm models have the most adaption with the experimental data. In the Langmuir model, the loading of AA on the adsorbent plane is monolayer and the tensile strength of all adsorption centers and sites is the same for the adsorbent component, meaning that AA molecules are uniformly located on the plane of the resin. Also, enhancing the temperature enables the increase of the uptake capability. In the Freundlich equation, it is assumed that multilayer penetration occurs on non-homogeneous planes or surfaces with different activity centers and positions in terms of energy and affinity with the adsorbent component. The R_L_ parameter at Langmuir's isotherm [Eq. (4)] indicates the desirability (0 < R_L_ < 1) of the adsorption process of AA by the adsorbent. In Freundlich's isothermal model, K_f_ increases with increasing temperature. Also, the amount of n higher than unity indicates that the M-KOH-RH adsorbent can be used for all AA concentration ranges. In the Temkin isotherm model, the values of the β parameter also increase with increasing temperature, which means more heat is absorbed. Also, the A_T_ parameter has the highest value in the isothermal isotherm model for the adsorbent at 35 °C, which indicates the adsorbent-adsorption interaction for the adsorbent at this temperature is larger than at the other temperatures. Examining the values of parameter which indicates the average free adsorption energy and the adsorption mechanism in terms of being physical or chemical. If the value of mean free energy is between 8 and 16 kJ/mol, the adsorption process is ion change type. If its value is less than 8 kJ/mol, it shows in adsorption that the mechanism of the adsorption process is physical^65^. In the D–R model, it is obvious that the physical adsorption mechanism (E > 8) is the dominant mechanism for adsorption^68^.

### Kinetic modeling

**Figure 13:**
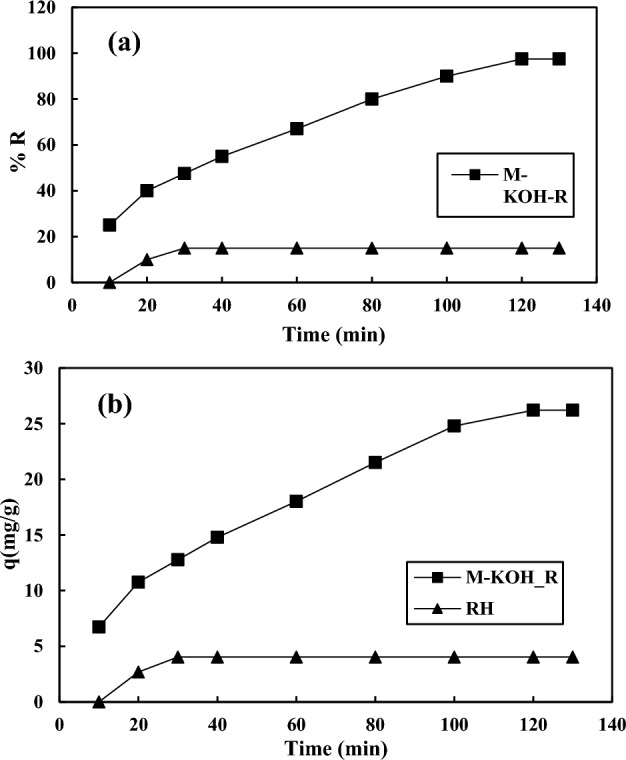
The diagram of (**a**) the adsorption percentage vs. the time, and (**b**) uptake capacity vs. the time.

In order to imply the physical or chemical mechanism between the resin and solute, the kinetic study of the adsorption is a useful and efficient method. Figure 13 shows the affinity of the adsorption percentage and uptake capacity to the time. According to this figure, the adsorption percentage and the adsorption capacity increase by time enhancement in both raw rice husk and the modified one. The equilibrium time for the rice husk and the modified rice husk has been observed at 120 and 30 min, respectively, which is owing to the penetration of AA between the layers of the adsorbents and AA deposited on the adsorbent surface. In the adsorption process, the active sites of the adsorbent become gradually altered and resulting in reduced adsorption rates^69,70^. It was concluded the modified rice husk could adsorb the AA rapidly. Adsorption kinetic data were obtained at the AA concentration of 481 ppm, and adsorbent dose of 0.898 mg using well-known kinetic models such as pseudo-first-order, pseudo-second-order, Elovich, and diffusion Intra-particle^71^. The kinetic equations are written below:

11 $$ {\text{The}}\;{\text{equation}}\;{\text{of}}\;{\text{pseudo - first - order}}\;{\text{model}}:{\text{ln}}\left( {{\text{q}}_{{\text{e}}} - {\text{q}}_{{_{{\text{t}}} }} } \right) = {\text{ln}}\left( {{\text{q}}_{{\text{e}}} } \right) - {\text{k}}_{{1}} {\text{t}} $$

12 $$ {\text{The}}\;{\text{equation}}\;{\text{of}}\;{\text{pseudo - second - order}}\;{\text{model}}:\;{\text{ln}}\left( {{\text{q}}_{{\text{e}}} - {\text{q}}_{{_{{\text{t}}} }} } \right) = {\text{ln}}\left( {{\text{q}}_{{\text{e}}} } \right) - {\text{k}}_{{1}} {\text{t}} $$

In the above equations, ${\mathrm{q}}_{\mathrm{e }}(\mathrm{mg}/\mathrm{g})$, and ${\mathrm{q}}_{\mathrm{t}}(\mathrm{mg}/\mathrm{g})$ indicate the uptake capability at the saturation state, and at the time of t, respectively. The constants of the pseudo-first-order and pseudo-second-order equations were denoted by ${\mathrm{k}}_{1}$ (1/min) and ${\mathrm{k}}_{2}$ (g/mg min(.

13 $$ {\text{The}}\;{\text{equation}}\;{\text{of}}\;{\text{Elovich}}\;{\text{model}}:q_{t} = \frac{1}{\beta }\ln \left( {\alpha \beta } \right) + \frac{1}{\beta }\ln \left( t \right) $$

14 $$ {\text{The}}\;{\text{equation}}\;{\text{of}}\;{\text{intraparticle}}\;{\text{diffusion}}\;{\text{model}}:\;{\text{q}}_{{\text{t}}} = {\text{k}}_{{\text{p}}} {\text{ t}}^{{{0}{\text{.5}}}} + {\text{C}} $$

In Eqs. (13) and (14), α and β are the constants of the Elovich model, which are called the adsorption rate (mg/g.min) and the desorption constant (g/mg), respectively. K_p_ (mg (g min^0.5^)^−1^) and C(mg/g) also describe the intra-particle diffusion rate and the boundary layer constant, respectively.

Table 10 shows the various parameters calculated by fitting four kinetic equations. According to Table 10, the pseudo-second-order equation has a more correlation relative to the other kinetic equations, which means that the chemical behavior is dominant in the separation procedure in which the electron sharing occurred between the composite and the solutes and also the intensity of separation of molecules on the adsorbent surface is linear and a function of the number of active sites. The intra-particle diffusion of adsorption took place in two stages, the first stage has a higher R^2^ and K_p_ than the second stage, which indicates that the dominant process is the mobility of AA from a liquid phase to the surface of the resin^72,73^.

**Table 10:** The kinetic constants of the different kinetic relations.

Kinetic model	Parameters	418 (mg/L)	600 (mg/L)	718 (mg/L)
Pseudo-first-order	q_e,cal_ (mg/g)	28.605	31.661	34.147
	k_1_ (1/min)	0.0263	0.0233	0.0221
	R^2^	0.9293	0.9641	0.9650
Pseudo-second-order	q_e,cal_ (mg/g)	36.496	45.248	49.019
	k_2_ (g/mg min)	5.22 × 10^–4^	2.91 × 10^–4^	2.96 × 10^–4^
	R^2^	0.9861	0.9553	0.957
Elovich	α (mg/g min)	1.519	1.396	1.661
	β (g/mg)	0.124	0.105	0.0981
	R^2^	0.9725	0.9419	0.9418
Intra-particle diffusion (step 1)	k_i1_	2.554	2.960	3.110
	I	− 1.2075	− 3.6659	− 2.550
	R^2^	0.9964	0.9815	0.9872
Intra-particle diffusion (step 2)	K_i2_	0.3297	0.1705	0.3297
	I	28.672	26.873	28.672
	R^2^	0.9649	0.9600	0.9649

### Adsorption thermodynamic

**Figure 14:**
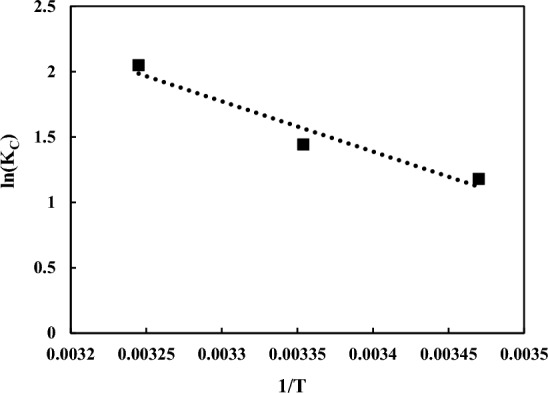
The diagram of ln(K_C_) vs. 1/T.

Thermodynamics of the separation process has been studied at various heat degrees to detect the impact of temperature on the separation procedure. The initial solution temperature provides the energy needed for the system and affects the system's adsorption capacity. In order to evaluate the behavior of each process in terms of feasibility and spontaneity, its energy functions and entropy should be considered^19,21,74^. Thermodynamic parameters like Gibbs free energy change (∆G°), enthalpy change (∆H°), and entropy change (∆S°) contribute to interpreting the adsorption performance under several heat degrees. For this aim, the quantities of ∆G°, ∆H°, and ∆S° at 293, 303, and 313 K were obtained as follows and their values have been calculated with respect to Fig. 14:

Where K_C_ is the saturation constant, R is the universal gas constant (8.314 kJ/mol K) and T is the absolute temperature (K). The thermodynamic factors of the adsorption have been listed in Table 11. This means that the process of adsorption of AA on the surface of the magnetic sorbent is endothermic, because the sign of ΔH° is positive. Positive values for ΔS^o^ indicate an increase of irregularity in the boundary layer between the solid phase and the liquid phase. Also, a low enthalpy value indicates weak interactions between AA and functional groups present on the adsorbent surface and the adsorption process is physical^65^. Finally, negative values for the ΔG° parameter mean that the ascorbic acid adsorption process on the solid surface is spontaneous^75^.

15 $$ {\text{ln}}\left( {{\text{K}}_{{\text{C}}} } \right) = - \frac{{{\Delta H}^{\circ} }}{{{\text{RT}}}} + \frac{{{\Delta S}^{\circ} }}{{\text{R}}} $$

16 $$ {\Delta G}^{\circ} = - {\text{RTln}}\left( {{\text{K}}_{{\text{C}}} } \right) $$

**Table 11:** The thermodynamic constants of the adsorption of AA on the modified RH.

Adsorbent	Temperature (°C)	K_c_	∆G° (kJ/mol)	∆H° (kJ/mol)	∆S° (kJ/mol K)
M-KOH-RH	15	3.254	− 2.825		
	25	4.231	− 3.573	31.972	120.253
	35	8.038	− 5.337		

### Adsorption mechanism

**Figure 15:**
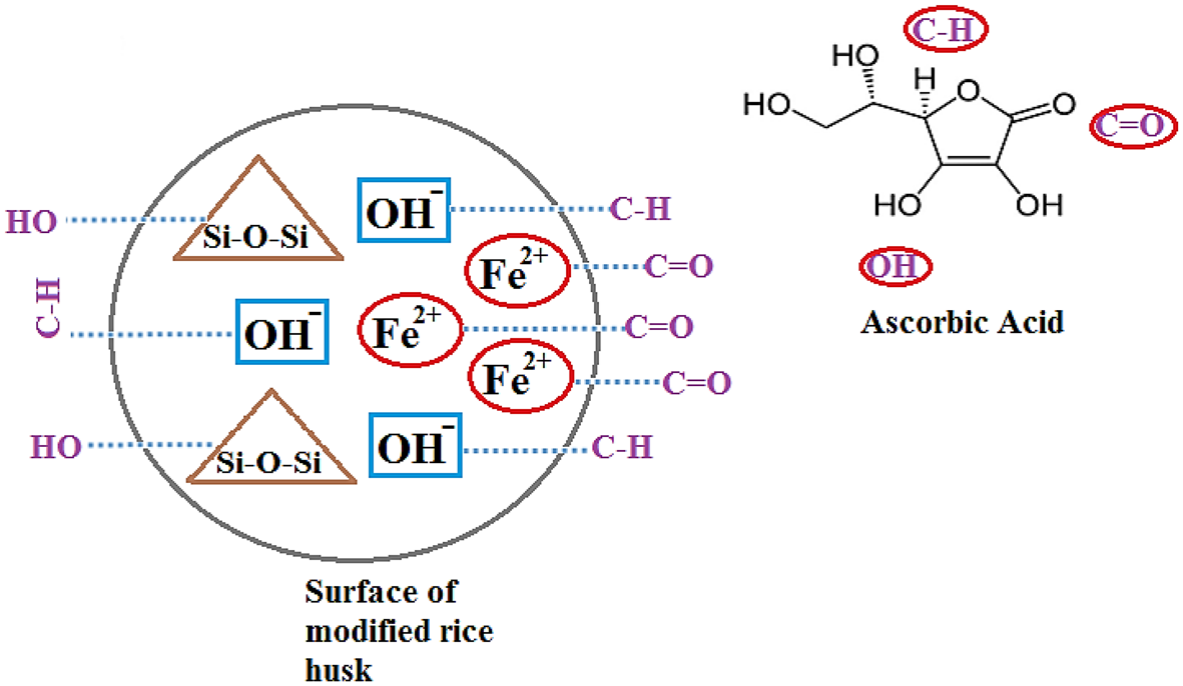
Schematic for the adsorption mechanism.

The mechanism for the adsorption of AA on the plane of the treated resin has been drawn in Fig. 15. With respect to this Scheme, electrostatic interactions and functional groups interfered with the adsorption mechanism of AA on the modified RH. The electrostatic interaction exists between the divalent iron ions and C=O in the aromatic ring of the AA and silicate ions of the adsorbent with the hydroxyl groups of the aromatic ring of the AA. In addition, the bond formation between the hydroxyl ions of the modified RH and the C–H of the AA backbone is the participation of the functional moieties in the separation mechanism. Moreover, the intra-particle diffusion equation in the kinetic study proves that the mass transfer mechanism of the AA adsorption divides into two parts including (a) Transportation of the AA particles from the AA solution onto the plane of the modified RH and (b) Penetration of AA molecules inside the channels of the modified RH. This kinetic model also explains that the first step or the first case is rapid. In addition, the separation mechanism of AA particles onto the plane of the resin is associated by releasing heat.

### Comparison

The comparison between the present work with the other works was collected in Table 12. This Table demonstrates that rice husk and modified rice husk were employed for the adsorption of different toxic components such as dyes, phenol, and metallic ions. According to this Table, both rice husk and modified rice husk have been used more for the adsorption of different dyes, and it was observed no papers about the adsorption of ascorbic acid onto the modified rice husk. Among the examined dyes, methylene blue could be adsorbed onto the modified rice husk (alginate/rice husk) with a higher uptake capacity (274.90 mg/g) relative to the other dyes. Rice husk could also adsorb the synthetic dye with a considerable adsorption percentage which was 99.50%. Besides its good uptake capacity in the adsorption of methylene blue, modified rice husk (KOH/rice husk) could likewise adsorb phenol with a high uptake capacity which was attained at 215.27 mg/g. The ability of adsorption for modified rice husk was relatively low for metallic ions like Mn(II) and Co(II) ions. Since the modified rice husk has exhibited good results in the elimination of dyes, phenol, and metallic ions, the authors decided to evaluate the potential of the modified rice husk (KOH/rice husk) for adsorption of ascorbic acid. Regarding this table, the modified rice husk has shown a dramatic adsorption percentage (92.936%) for ascorbic acid, therefore, KOH/rice husk can be a suitable natural adsorbent for adsorbing the ascorbic acid.

**Table 12:** Comparison the present work with the other studies.

Researcher	Adsorbent	Component	%R or q (mg/g)				Ref.
Chen et al.	Sludge-rice husk biochar	Four dyes (DR, AO, RB, MB)	q:DR	AO	RB	MB	^76^
			37.92	23.61	29.43	13.75	
Kheddo et al.	Rice husk	Synthetic dyed	R = 99.50%				^77^
Alver et al.	Alginate/rice husk	Methylene blue	q = 274.90				^78^
Tavlieva et al.	Rice husk	Mn(II)	q = 18.01				^79^
Anbia et al.	Carbon mesostructured	Ascorbic acid	q = 39.00				^80^
Lv et al.	KOH/rice husk	Phenol	q = 215.27				^81^
Suc and Chi	Microwave activated rice husk ash	Rhodamine B	q = 21.89				^82^
Ashrafi et al.	NaOH-modified rice husk	Methylene blue	R = % 97.66				^83^
Dison et al.	Rice husk modified by ultrasound	Co(II)	q = 35.00				^84^
Foroutan et al.	Walnut shell ash/starch/Fe_3_O_4_	Cu(II)	q = 45.40				^19^
Savari et al.	Zeolite–zirconium	Fluoride	q = 32.98				^22^
Ahmadi et al.	Montmorillonite clay/starch/CoFe_2_O_4_	MV, MB	MV		MB		^20^
			q = 43.95		q = 47.51		
Foroutan et al.	ZIF-8-chicken beak hydroxyapatite	Ni(II)	q = 63.49				^21^
Gong et al.	Shellac-coated iron oxide	Cd(II)	q = 18.80				^52^
Zhang et al.	CuFe_2_O_4_/activated carbon composite	Acid Orange 7	q = 392				^85^
Mathurasa and Damrongsiri	Modified rice husk	Nitrate	q = 25				^86^
Saremi et al.	Date palm leaves	Tetracycline	q = 76.92				^87^
Kaur et al.	Modified rice husk	Imazethapyr	q = 166.51				^88^
Kaykioğlu et al.	H_2_SO_4_-activated rice husk	Methylene blue	q = 44.25				^89^
Ghaemi et al.	dolomite powder	Cd(II), Ni(II)	Cd (II)		Ni(II)		^90^
			q = 1.46		q = 1.70		
Present work	KOH/rice husk	ascorbic acid	R = % 92.936				–

### Reusability

**Figure 16:**
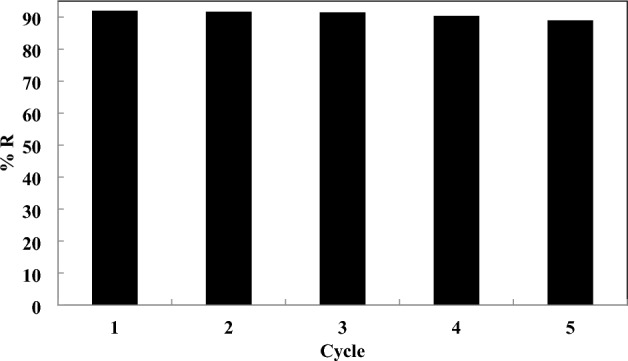
The reusability result for the modified RH at the acid concentration of 486.929 ppm, adsorbent dosage of 0.875 g, and time of 105 min.

In order to save adsorbents, it is essential to survey the reusability of modified RH during adsorption. The reusability of the sorbents is an important factor in industrial applications. In our research, 0.1 mol/L HCl has been selected for leaching the AA from the modified RH^68^. In fact, the modified RH with the adsorbed AA molecules was soaked in the vessel containing 0.1 mol/L HCl for 30 min, and then the adsorbent was rinsed with the deionized water 3 times. Subsequently, it was dried in the oven. After this mentioned process, the modified RH was used again for trapping the AA molecules. The results of the desorption prove that the modified RH is a suitable adsorbent for leaching the AA molecules because the adsorption efficiency at the equilibrium point in the initial and the terminal cycle has been detected at 92 mg/g and 89.5 mg/g, respectively (Fig. 16). In other words, the adsorption efficiency of the modified RH decreased % 2.71 only. Hence, the modified RH is advised to use more than five times without a dramatic reduction in the adsorption efficiency, implying that the modified RH is completely reused frequently.

### Effect of cation and anion

**Figure 17:**
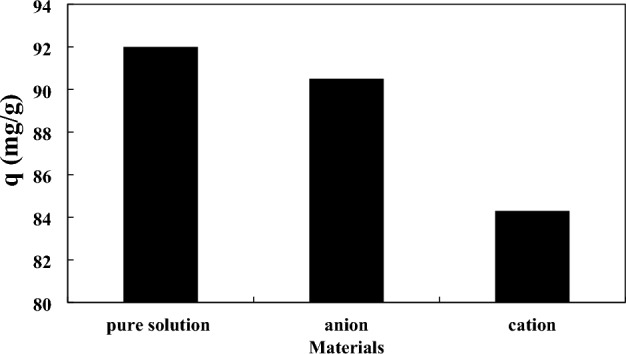
The effect of anion and cation on the adsorption capacity of the AA using the modified RH at the acid concentration of 486.929 ppm, adsorbent dosage of 0.875 g, and time of 105 min.

In this section, we want to know how the anion and cation alter the adsorption of AA. The results are exhibited in Fig. 17. The cation and anion in this research are Ca^2+^ and Cl^−^ With respect to Fig. 17, the presence of an anion has a slight impact on the adsorption capacity, and the presence of a cation has almost a considerable effect on the adsorption capacity. The adsorption capacity of AA on the magnetic RH without the presence of cation and anion has been obtained at 92 mg/g. The adsorption capacity of AA on the magnetic RH in the presence of cation and anion has been obtained at 84.3 and 90.5 mg/g, respectively. Since the sorbent has a negative charge and anions have also a negative charge. Therefore, the repulsion electrostatic force is created between the anions and the surface of the adsorbent which has a little impact on the adsorption of AA onto the adsorbent. In addition, when the cations are added to the AA solution, they compete with the AA particles for binding with the functional groups. Besides, cations can bind with the negative sites of the adsorbent surface and the negative charge of the AA structure^91^.

## Conclusion

In this current research, the RH was modified by adding Fe_2_O_3_ to its network for the adsorption of AA from the aqueous solution for the first time. The characterization tests, isotherm, kinetic, thermodynamic modeling, and reusability test have occurred for studying the adsorption potential of the modified RH. Besides the experimental results, the design-expert software version 11 was considered for detecting the optimum experimental conditions in which the adsorption efficiency would be maximized at those conditions. These results have been derived which were written below:

1. The optimum conditions of the design-expert software have been obtained at 486.929 ppm of acid concentration, 0.875 g of the adsorbent dosage, and 105.397 min of the contact time, and the adsorption efficiency in these conditions was determined at 92.936%.
2. The reusability test exhibits that the adsorption efficiency at the equilibrium point in the initial and the terminal cycle has been detected at 92 mg/g and 78 mg/g, respectively, which showed the modified RH can be used more than five times without dramatic reduction in the adsorption efficiency.
3. The surface area of the RH and modified RH was determined at 98.17 and 120.23 m^2^/g, respectively.
4. The thermal stability of the modified rice husk was 450 °C.
5. The Langmuir model had the highest R^2^ of 0.9982, 0.9996, and 0.9985 at the temperature of 15, 25, and 35 °C, respectively, and also the q_max_ values in these temperatures have been calculated at 19.157, 31.34, and 38.75 mg/g, respectively.
6. The pseudo-second-order kinetic model had the best agreement with the experimental results. In this kinetic model, the values of q have been measured at 36.496, 45.248, and 49.019 mg/g at the acid concentration of 418, 600, and 718 ppm, respectively.
7. The values of ΔH° and ΔS° were measured at 31.972 kJ/mol and 120.253 kJ/mol K, respectively, which proves the endothermic and irregular nature of the adsorption of AA.
8. Electrostatic interactions and functional groups interfered in the adsorption mechanism of AA on the modified RH.

## Data Availability

The datasets used and/or analyzed during the current study available from the corresponding author on reasonable request.
